# Tectonic blocks and molecular clocks

**DOI:** 10.1098/rstb.2016.0098

**Published:** 2016-07-19

**Authors:** Kenneth De Baets, Alexandre Antonelli, Philip C. J. Donoghue

**Affiliations:** 1 School of Earth Sciences, University of Bristol, Life Sciences Building, Tyndall Avenue, Bristol BS8 1TQ, UK; 2 GeoZentrum Nordbayern, Fachgruppe PaläoUmwelt, Friedrich-Alexander-Universität Erlangen-Nürnberg, Loewenichstr. 28, 91054 Erlangen, Germany; 3 Department of Biological and Environmental Sciences, University of Gothenburg, Box 461, 405 30 Göteborg, Sweden; 4 Gothenburg Botanical Garden, Carl Skottsbergs gata 22A, 413 19 Göteborg, Sweden

**Keywords:** molecular clock, calibration, fossil record, biogeography, tectonics

## Abstract

Evolutionary timescales have mainly used fossils for calibrating molecular clocks, though fossils only really provide minimum clade age constraints. In their place, phylogenetic trees can be calibrated by precisely dated geological events that have shaped biogeography. However, tectonic episodes are protracted, their role in vicariance is rarely justified, the biogeography of living clades and their antecedents may differ, and the impact of such events is contingent on ecology. Biogeographic calibrations are no panacea for the shortcomings of fossil calibrations, but their associated uncertainties can be accommodated. We provide examples of how biogeographic calibrations based on geological data can be established for the fragmentation of the Pangaean supercontinent: (i) for the uplift of the Isthmus of Panama, (ii) the separation of New Zealand from Gondwana, and (iii) for the opening of the Atlantic Ocean. Biogeographic and fossil calibrations are complementary, not competing, approaches to constraining molecular clock analyses, providing alternative constraints on the age of clades that are vital to avoiding circularity in investigating the role of biogeographic mechanisms in shaping modern biodiversity.

This article is part of the themed issue ‘Dating species divergences using rocks and clocks’.

## 1. Introduction

Establishing an evolutionary timescale for the tree of life is a focal yet elusive goal of evolutionary biology. The fossil record has traditionally provided the timescale for evolutionary history and, while its imperfections are widely appreciated, it remains the principal means by which its successor, the molecular clock, is calibrated to time. Since its conception half a century ago [1,2], the application of molecular clock methodology has undergone extensive development, particularly to account for variation in the rate of molecular evolution among lineages and, more recently, to accommodate the inaccuracies and imprecision inherent in the use of fossil evidence in calibration [3–8].

Given that the molecular clock was developed explicitly to overcome the incompleteness of the fossil record, it is ironic that fossil evidence remains the literal rate-determining step in molecular clock analyses [8]. Fossils can only provide minimum time constraints on the age of clades because the earliest representatives of evolutionary lineages may lack diagnostic characteristics and their chances of fossilization are low. However, molecular clocks must be calibrated by estimates of divergence timing and so it has become necessary to provide a probabilistic judgement of the degree to which fossil minima approximate divergence timing. The established means of achieving this could be considered a dark art [9]. There are numerical approaches to estimating divergence timing based on fossil stratigraphic data [10–13], diversification modelling of fossil and extant lineages [14–16], or integrated analysis of morphological and molecular datasets and evolutionary models. Each of these approaches allows fossil species to be integrated into divergence time analyses and provide calibration directly [17–19]. However, they are often data-intensive and laborious, and, therefore, commonly passed over in favour of phylogenetic bracketing [5,20], or applying probability functions that express some visceral perception of the degree to which fossil minima approximate the true time of divergence [21]. Sensitivity studies show that such arbitrary ‘time priors' (prior assumptions about the age of a lineage) impact heavily on divergence time estimates—weight that is undue given the paucity of evidence on which they are invariably based [7,22,23].

Thus, critics have advocated that fossil-based calibration should be supplemented or abandoned entirely in favour of calibrations that are based on geological events that have influenced the diversification and distribution of taxa [9,24–26]. Such events include everything from the fragmentation and assembly of supercontinents and the opening and closure of vast oceans, to the formation of volcanic islands, land bridges, salt barriers, mountains, lakes and changes in river drainage patterns [27]. Advocates of biogeographic calibration argue that tectonic calibrations are more accurate and reliable than fossil-based calibrations because they can directly evidence both maximum and minimum constraints on the age of lineage divergence events. Tectonic events can be dated geochronologically using radiometric dating and magnetostratigraphy [28,29] with a level of precision that greatly surpasses most fossil-based constraints. Based on the distribution of living lineages, tectonic calibrations can be used to establish an evolutionary timescale for groups with a poor fossil record or none at all. Thus, although the molecular clock has conventionally been employed in groups with a good fossil record to assess the efficacy of the fossil record, tectonic calibrations allow us to realize the original but forgotten aim of the molecular clock—to establish an evolutionary timescale for lineages lacking an appreciable fossil record [3] or for which no other means of direct calibration exists [30–36].

It should come as no surprise, therefore, to discover that biogeographic calibrations have been adopted widely [27], particularly, in terrestrial groups with a poor fossil record. However, their accuracy and precision flatters to deceive. As we go on to show, biogeographic calibrations are subject to many of the errors associated with fossil-based calibrations, and introduce a number of additional artefacts. Effectively accommodating these errors renders biogeographic calibrations more accurate, but often less precise.

## 2. Constraining biogeographic calibrations

**Figure 1.**
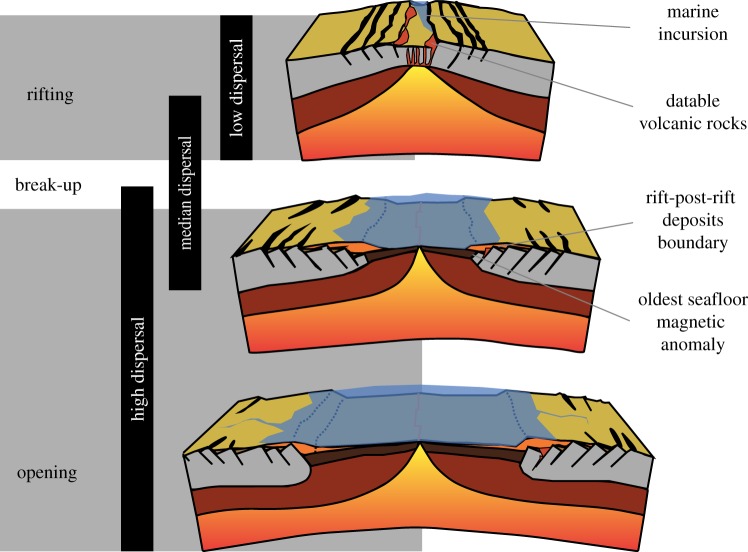
Possible relationship between divergences of terrestrial groups with different dispersal abilities and the age constraints from the break-up of continents and formation of oceans. Continent reconstructions are simplified after Stanley & Luczaj [37].

Episodes of continent fragmentation, collision and uplift are protracted, and dating is constrained by multifarious, invariably conflicting lines of geological evidence. Each of these lines of evidence has its own suite of dating uncertainties that can belie the accuracy of precise biogeographic calibrations. The main geological methods used for dating the break-up of continents (figure 1) are as follows.

### (a) Radiometric methods

Igneous rocks can be dated very precisely with radiometric methods and, depending upon the interpretation of their spatial and temporal relationship, they can be used to constrain the timing of continent fragmentation, collision or uplift [28]. The eruption of continental flood basalts, for example, has often been linked temporally and spatially with continental break-up [38]. In some cases, igneous rocks are restricted to the post-break-up phase and the age difference between basalt emplacement and break-up (oldest ocean crust magnetic anomaly) may vary between 3 and 35 Myr [39].

### (b) Stratigraphic methods

The establishment or disappearance of a marine barrier can be dated more directly at the transition from terrestrial to marine sedimentary facies (position of the coastline) or vice versa. However, their age can usually only be established indirectly, using stratigraphic correlation to directly dated sequences elsewhere [6,8]. The oldest synrift and youngest post-rift deposits constrain the timing of break-up [28]. Their stratigraphic relationship is not conformable and can correlate more or less well with the oldest oceanic seafloor [39]. The start of rifting is even more difficult to date, relying on rift unconformities or the presence of sediments or igneous rocks indicative of extensional tectonics [28,29]. Geochemistry, sedimentary facies and fossil content can constrain not only the establishment, but also the magnitude, of dispersal barriers.

### (c) Palaeomagnetic methods

These rely on the palaeomagnetic signal recorded in rocks. The oldest seafloor magnetic anomaly is often used as proxy for continent fragmentation, but postdates the initial stages of rifting and even so, the earliest ocean crust is invariably buried beneath post-rift sediments, frustrating direct dating [28]. Undeformed seafloor is absent from the Palaeozoic [40,41], and a well-calibrated geomagnetic timescale is available from the Middle Jurassic [42]. Magnetic anomalies are considered confidently dated only if they occur outboard of the ocean–continent transition and are present across the continent margin and on both sides of the rift [38,43,44]. Thus, the pattern of magnetic anomalies is mostly measured at sea and correlated with one of the many competing ocean–continent magnetic polarity timescales [29,45]. Dating precision is diminished by long periods without magnetic polarity change, such as the 43 Myr Cretaceous superchron [46].

## 3. Precision without accuracy

Despite the widespread need for alternative calibrations, biogeographic calibrations have not enjoyed the same scrutiny [27,47] and, therefore, methodological development as have fossil-based calibrations [6–8,48]. Biogeographic calibrations rely on a number of explicit and implicit assumptions that have associated errors which must be accommodated to ensure calibration accuracy. These include, first and foremost, the assumption that a specific geological event is causal to the biogeographic event that underpins the cladogenesis or distribution of descendent species. Although this is the foundation of any biogeographic calibration, it is rarely justified or evidenced. This is unfortunate because it is widely appreciated that extant distributions need not reflect the historical range of a lineage. Thus, lineages are not necessarily as old as the geological terranes that their living representatives inhabit [49,50]; and they could, in fact, be older if they originated elsewhere [51]. The geographical range of species can change considerably on geological timescales as a consequence of dispersal, localized extinction, climatic and environmental changes, and the evolution of the species' intrinsic environmental tolerances (niche conservatism or lability). Environmental controls on the geographical range of species can lead to biogeographic convergence—pseudocongruence—between lineages of very different antiquity [52].

The dating of historical biogeographic events used in divergence time estimation has an equally poor record of justification [27,53]. While a radiometric date may provide an accurate and precise estimate of the age of its source rock, it is seldom considered how this age reflects the timing of a geological event [28]. Biogeographic calibrations are commonly used in molecular dating as events of short duration, or as a specific date, often with a small, if any, associated error. However, the most popular biogeographic calibrations are tectonic affairs in which landmasses fragment (reducing or preventing gene flow in hitherto inter-breeding terrestrial populations), or collide (enabling gene flow in terrestrial populations that were previously genetically isolated). Even for geological events considered 'unusually fast', such as the collision of the Indian subcontinent with Asia (e.g. [54]) and the Messinian crisis [55], in the vast majority of cases these events occurred over protracted episodes and not at finite points in geological time. There is almost never a single, unequivocal palaeogeographic reconstruction, and competing models are based on different methods and multiple lines of frequently conflicting evidence. For instance, palaeo-coastlines may be established simply by averaging the distance between the extent of coeval marine and continental sedimentation. Differing approaches can be employed to accommodate missing data (e.g. areas of non-deposition or erosion), such as maximizing or minimizing sea and land areas [56,57]. These factors make it difficult to constrain both the role of biogeographic events and the timing of their effect in driving lineage divergence. Palaeogeographic reconstructions are created to present a consensus for a geological time interval, not a finite point in time [29,41,53]. Thus, they are often interpreted too literally and causally by biologists. Although geological events like continent fragmentation and ocean closure impact in different regions at different times [29], most biological studies simply use an estimate of the start or end date of tectonism as a basis for priors on lineage divergence timing [26].

Finally, most studies assume implicitly that geological constraints on the timing of lineage divergence are equivalent in all organisms, ignoring taxon-specific differences in their environmental tolerance, ecological requirements and dispersal ability. These differences may in turn also be a function of the magnitude of the barrier(s) or landbridge(s) over time and get influenced, e.g. by the distance and depth of seaways between continents [58]. Bearing this in mind, it is clear that geological events will impact different ecological groups at different times. For instance, the very initial stages of continental rifting, which can last for several tens of millions of years [28,29], may prevent gene flow among highland-adapted [59] and salinity-intolerant amphibians (e.g. pipid frogs), yet populations of pelagic birds can maintain gene flow even across broad oceans. The same biogeographic episode may be causal in lineage divergences in both highland amphibians and pelagic birds, but it does not follow that the same time prior should be used for dating both groups. Similarly, when landmasses connect, the first events of biotic interchange should be expected from easily dispersed and environmentally generalist organisms. The relationship might be reversed or even more complex for marine organisms.

In sum, biogeographic calibrations are subject to at least as many uncertainties as are fossil-based calibrations. Some of these are the same, including the problems associated with dating geological sequences and the degree to which those geological dates approximate lineage divergence [8,60,61]. However, biogeographic calibrations introduce further uncertainty, not only in terms of the degree to which the geological date approximates the timing of the biogeographic event, but also the question of whether the geological event was indeed causal to lineage divergence, vicariance or dispersal of the focal taxon [27,47]. This means that biogeographic calibrations, as they are currently conceived, provide precision without accuracy. We, therefore, need formal criteria for establishing biogeographic constraints in a manner that accommodates the attendant uncertainties, and then reflect these uncertainties in probabilistic priors on clade ages. Biogeographic calibrations abound with uncertainties, but they need not be fatal for the approach of calibrating divergence times using biogeographic constraints.

## 4. Accommodating error in biogeographic calibrations

To accurately reflect the uncertainty over the timing of lineage divergence events, biogeographic calibrations must be implemented as probabilistic constraints that entertain (i) the probability that a geological event was causal to the calibrating node, (ii) errors in the accuracy of dating the geological event, (iii) the temporal and spatial extent of the barriers associated with the geological event, and (iv) the differential impact of the geological event on organisms with different ecologies. Above all, biogeographic calibrations must be reproducible so that, like all other assumptions in divergence time estimation, they may be scrutinized for veracity. Furthermore, since the evidence on which they are based remains in flux, it must be possible to determine the impact of changes to component variables, from the redating of rocks, through revolutions in phylogenetic hypotheses, to the revision of the geological timescale.

The most crucial step in establishing a biogeographic calibration is the first—justifying the role of a geological event in causing a lineage divergence. Ideally, this should be based on independent geophysical evidence, e.g. avoiding palaeogeographic reconstructions inferred from biological data. Maintaining such a distinction allows palaeobiogeographic data to be put to work, complementing modern biogeographic data and, indeed, providing a unique test of whether modern biogeography is a reflection of phylogenetic history, discriminating instances of pseudocongruence [52].

Traditionally, biogeographic hypotheses have been tested with parsimony-based cladistic or event-based methods [62–64], which were not designed to incorporate information on the absolute timing of the diversification of lineages. Modern parametric biogeographic approaches, based on maximum-likelihood and Bayesian algorithms, permit the inclusion of divergence time estimates, as well as external lines of evidence, such as information on past climate and geography, the fossil record of a lineage or its ecological tolerance [65].

When it is possible to justify a causal role for a geological event in lineage divergence, its age interpretation should also be justified explicitly, drawing on primary geological evidence. In instances where age evidence relies on relative dating techniques (e.g. magnetostratigraphy, lithostratigraphy and biostratigraphy), errors associated with stratigraphic calibration must also be accommodated. This entails a process of correlation between sections, sometimes through a daisy chain of multiple intermediate steps, until it reaches a section in which time-equivalent strata have been directly dated, or in which biostratigraphic, magnetostratigraphic or other markers occur that have already been calibrated to absolute geological time [8,60]. At each correlative step, a minimum and maximum age interpretation is possible and it is necessary to follow the most conservative interpretation of the age evidence. Deciding among the alternatives depends on whether the data are to be used in establishing a minimum, maximum or combined temporal constraint. Even more elaborate priors on the timing of lineage divergence could be implemented based on geological models, just as in the establishment of fossil-based temporal constraints [8].

Assuming that it is possible to date a geological event accurately (which will vary from case to case, e.g. with events linked to volcanic rocks being generally the most reliably dated), it remains necessary to estimate the degree to which the event affected lineage divergence. Two principal factors must be entertained. First, the protracted nature of some of the geological ‘events’ that inspire calibrations, such as the opening of the Atlantic, have differential geographical effects at different times in different regions. As magnetostratigraphy demonstrates clearly, the first major phase of continental fragmentation in the opening of the Atlantic was between North America and North Africa, creating a proto-Caribbean, but with effectively continuous landmasses to the north and south. The complete north–south opening of the Atlantic took a further 80–100 Myr [29]. The second principal consideration is the ecological impact of such geographical change, such that temporal constraints on tectonic events will be more or less limiting on gene flow. Thus, temporal constraints on geological events must be interpreted for organisms with different or evolving ecologies and geographic ranges.

We provide examples of how these principles might be implemented, particularly for terrestrial lineages, in establishing biogeographic calibrations based on the uplift of the Isthmus of Panama, bridging North and South America, and two of the most widely employed biogeographic events associated with the fragmentation of the Pangean supercontinent: the separation of New Zealand from Gondwana, and the opening of the Atlantic Ocean.

## 5. Uplift of the Panama Isthmus and closure of the Central American Seaway

The bridging of North and South America triggered not only a spectacular interchange of terrestrial biota [66], but also the relative isolation of Atlantic and Pacific tropical marine biota. For decades, the timing of this geological event was considered one of the best dated of all vicariance events [67]. Consequently, numerous studies adopted the universally accepted approximately 3.5 Ma date for the uplift of the Isthmus of Panama to calibrate molecular phylogenies [68]. Recently, however, substantial and independent lines of evidence based on magmatic cooling, U/Pb dating, magnetostratigraphy, neodymium isotopic data, detrital zircons and molecular divergence times suggest that the uplift of the Panama Isthmus and the Great American Biotic Interchange were considerably more complex and protracted episodes than traditionally assumed, beginning already some 23–25 Ma [68–74].

Could molecular phylogenies be dated based solely on these new geological reconstructions? This will depend heavily on the focal organism and the assumptions made. For instance, even though the Central American Seaway—the main aquatic barrier separating the South American plate and the Panama arc—closed by *ca* 13–15 Ma, shallow and transient channels probably existed west of the canal area [72,73]. To further complicate matters, biotic dispersal may have been influenced by climatic and environmental changes [75], which might explain why some taxa—such as most mammals—did not begin to cross the Isthmus region in substantial numbers until the last 3–4 Ma [68,76,77].

Any use of the geological reconstruction for the Panamanian Isthmus should accommodate this complexity. For a randomly selected phylogeny, this could mean designing age priors reflecting the empirical patterns estimated from cross-taxonomic biogeographic analyses, rather than following the ages from strictly geological models. Following recent studies [68,77,78], this would probably mean increasing prior likelihoods for node ages separating South and North American terrestrial disjunctions at 20 and 6 Ma, whereas, for a shallow-marine clade comprising an Atlantic/Pacific disjunction, the likelihood of vicariance should increase at *ca* 24 and 9 Ma.

**Figure 2.**
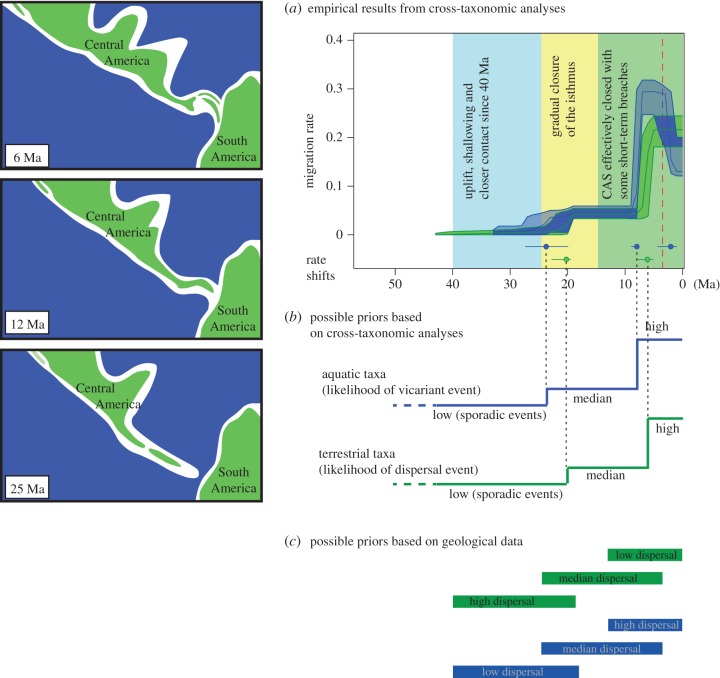
(*a*,*b*) History of the Panama Isthmus (adapted from [69]), its relationships with dispersal time estimates (adapted from [68]) as well as different proposals for implementing priors for terrestrial (green) and shallow-marine (blue) taxa. The red dashed line indicates the generally assumed timing of the Isthmus closure (*ca* 3.5 Ma). The upper two priors (*b*) are based on likelihood of dispersal and vicariant events based on cross-taxon analyses (in (*a*)). The lower two are based on the potential relationship between the geological data on the establishment of the Panama Isthmus (listed in (*a*)) and divergence in groups with different dispersal abilities. If no clear link between environmental changes and/or dispersal abilities can be established, it might be the more adequate to use a uniform prior across all intervals.

To increase accuracy (albeit reducing precision), the relative likelihoods of calibrated nodes and their confidence intervals could also be designed according to empirical estimations, even if simplified into discrete intervals and simpler functions such as uniform distributions (figure 2). To avoid circularity, it is crucial that the focal taxon is not also used in the estimation of those priors.

## 6. Separation of New Zealand from Gondwana (Antarctica, Australia)

**Figure 3.**
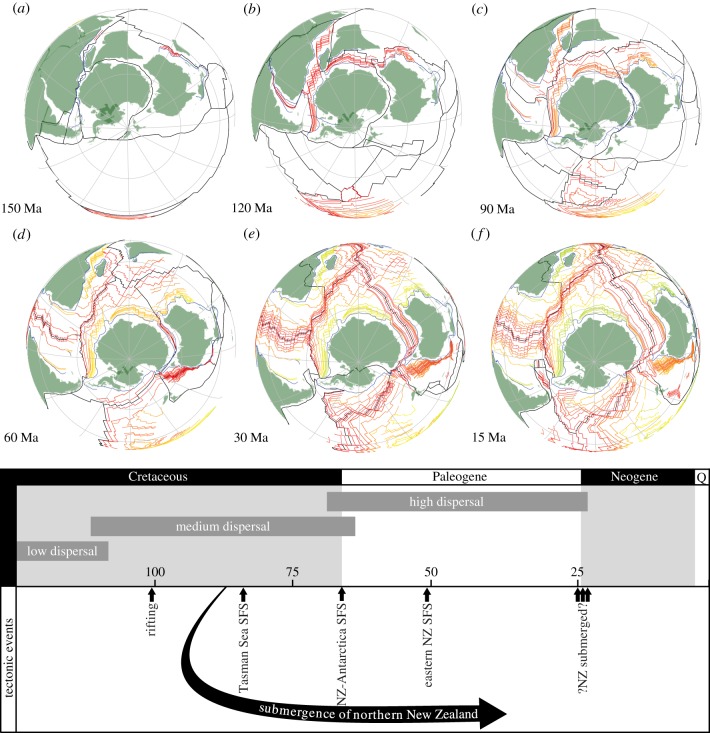
(*a*–*f*) Different phases in the history of New Zealand (adapted from [29]) with possible links to divergence for terrestrial groups with different dispersal abilities.

The timing of the separation of New Zealand from Gondwana has been used to constrain divergence time analyses of a wide range of organisms [27], from plants [79], velvet worms [80] and insects [81], to amphibians [82–84] and, surprisingly, flying birds [31,32,85–93]. New Zealand is part of the largely submerged continent Zealandia that, through the opening of the Tasman Sea, rifted from Antarctica and Australia in the Cretaceous (figure 3; [57]). Almost invariably ([94] for an exception), a single date or short time span has been used to calibrate lineage divergences in which this vicariance episode is implicated, based on the oldest magnetic anomalies in the Tasman Sea (80–82 Ma). None have integrated all of the associated uncertainty.

Rifting is believed to have started in the latest Albian (approx. 100 Ma), evidenced by direct dating of ashes at an unconformity separating older, subduction-related rocks from younger, less-deformed strata (101.6 Ma ± 0.2 Myr; [95]). A widespread break-up unconformity overlain by sediments of Late Santonian age (approx. 83.6 Ma) and seafloor spreading in the New Zealand region is interpreted to have begun by Chron 34y (83.64 Ma), the oldest magnetic anomaly identified in the central Tasman Sea [96]. The oldest reliably identified seafloor southeast of New Zealand was formed during Chron 33r (79.90 Ma; [97]). However, there is evidence for Chron 34 (125.64–83.64 Ma) adjacent to the Campbell Plateau, in the oldest oceanic crust between southeastern New Zealand and Antarctica [98]. Nevertheless, the rifting of New Zealand and Australia was progressive, extending from south to north, leaving these continents connected until the end of the Cretaceous, or even later [99]. Rifting at the eastern margin stopped at 52 Ma with the start of seafloor spreading (Post-Chron 24 [96]). The isolation of the New Zealand region from Gondwana is also associated with marine submergence. Marine sedimentation occurred over large areas of northern New Zealand from 87 to 85 Ma [95]; however, it is not clear whether New Zealand was emergent throughout the Middle Cenozoic (22–25 Ma), perhaps, leading to a loss of continental life during periods of submergence [57]. It would, therefore, be best to use a uniform prior with soft bounds ranging from 101.8 to 22 Ma (or even 0 Ma), which would however lead to a loss of precision (figure 3).

The New Zealand–Gondwana case should be used for calibration with caution. It is particularly difficult to justify for flying and otherwise vagile organisms that could have colonized New Zealand long after rifting and its partial or entire submergence [100]. Conversely, while a marine or terrestrial connection might have existed between subcontinents, endemicity might have been established by distinctive climate or other palaeoenvironmental factors. This example illustrates that at least as much work should be done to disentangle the geological constraints on the establishment or disappearance of ecological barriers, as is done to obtain sequence data.

## 7. Opening of the Atlantic Ocean

**Figure 4.**
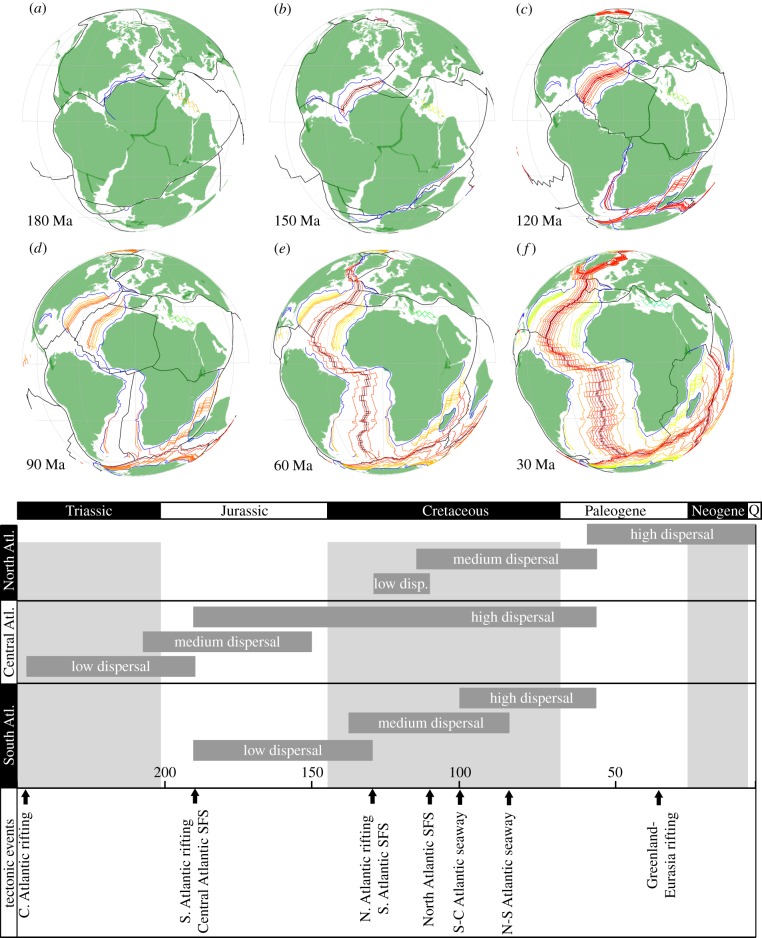
(*a*–*f*) Different phases in the opening of the Atlantic Ocean (adapted from [29]) with possible links with divergences of terrestrial groups with different dispersal abilities. Note that just oceans and continents are drawn; these have no bearing on coastlines which are more land-inward and more difficult to reconstruct.

Many terrestrial sibling lineages exhibit a pattern of distribution compatible with vicariant divergence caused by the opening of the Atlantic Ocean, as part of the fragmentation of Pangaea. Thus, the opening of the Atlantic is one of the widely employed biogeographic calibrations in divergence time estimation for plants [35,101,102], onychophora [80], insects [81,103,104] and amphibians [83,84,105]; by some it is also considered to be among the best-constrained temporally [106]. However, the opening of the Atlantic was a protracted process and the physical separation of the continents was not synchronous along the line of rifting, with seafloor spreading beginning in the Central Atlantic before propagating north from the southernmost Atlantic (figure 4), and finally, extending into the northernmost Atlantic [29]. The timing of different events (establishment of a rift valley, establishment of a seaway, start of seafloor spreading, etc.) within this tectonic episode remains contentious, not least since they draw upon many different sources of evidence from different geographical regions.

Initial continental rifting, evidenced by the establishment of extensional basins, was initiated by the Middle Triassic in the Central Atlantic (Anisian: 247.1–237 Ma; [107]), by the Early Jurassic (190 Ma) in the southernmost South Atlantic [29], propagating north by the Late Jurassic [38,108]. The major phase of rifting in the North Atlantic is evidenced by synrift Barremian/Late Hauterivian age sediments (130–125 Ma) deposited in extensional basins [29,109].

The establishment of seafloor spreading occurred later. In the Central Atlantic, where it marks the initial break-up of the Pangaean supercontinent, age estimates range from 200 to 170 Ma [29] depending on the interpretations of magnetic anomalies and sedimentary breaks on tectonically passive continental margins. Some models [110] invoke an early ridge jump at 170 Ma, rather than significant spreading asymmetry, to account for increased crustal accretion onto the North American plate, while other models suggest a diachronous opening [111] with the beginning of break-up already in the latest Rhaetian at about 200 Ma [112]. This might not have extended into the southernmost North Atlantic until about 185 Ma [111], or even 170 Ma (based on the age of the oldest drilled continental crust [112]), but recent studies suggest that seafloor spreading was initiated by 190 Ma (and certainly not before 203 Ma) based on magnetic data and dating of salt basins offshore Morocco and North America [113].

South Atlantic rift onset unconformities have been dated within the range 220–129 Ma [114], and break-up unconformities to 136.1–130.77 Ma (Late Valanginian to Barremian; [39]) for the southern segment. Seafloor spreading is presumed to have started in the Falkland segment by 133.8 Ma (M10; [43]), however, the oldest magnetic anomalies in the southern segment indicate spreading initiated within the interval 133.40–130.60 Ma [29,43]. The Central segment is poorly dated because spreading was initiated during the Early Cretaceous magnetic quiet time (Cretaceous magnetic superchron; Torsvik *et al*. [38]; Moulin *et al*. [43]). However, this can be constrained on the timing of opening north of the Walvis Ridge, Rio Grande Rise, dated on the cessation of salt deposits in the large evaporitic basins that mark the final stages of rifting at around the Aptian–Albian boundary (113 Ma). Seafloor spreading might have propagated into the Equatorial segment of the Southern Atlantic by 125.93 Ma (after magnetic anomaly M0), but most estimates suggest this occurred later, by 120.4 Ma [45,108], 113 Ma (Aptian–Albian; Moulin *et al*. [43]), 100 Ma [38] or 102–96 Ma [114]. Nevertheless, only a small ocean basin might have separated the southernmost part of South America and South Africa and Brazil during this time (cf. [115]). Until the Albian–Cenomian (*ca* 100 Ma), Brazil might have remained in close proximity with equatorial and southernmost Africa [29,38,46]. Largely symmetric spreading occurred along the entire length of the South Atlantic from anomaly C34 onwards (from 83.64 Ma). An open marine connection to the Central Atlantic oceans did not occur until the Late Albian (107.59–100.5 Ma), through narrow but locally deep basins [116]. North Atlantic–South Atlantic deep-water circulation was established between 95 and 83.6 Ma (Late Santonian; [46,117]). However, there remains evidence of connections between North America and Eurasia well into the Cenozoic [118], including the North Atlantic or Thulean Land Bridge through Greenland–Iceland–Faeroe–Scotland, the De Geer Land Bridge across the Norwegian–Greenland Sea, and the North America–Eurasia or Bering(ian) land bridge.

Seafloor spreading propagated from the Central Atlantic into the North Atlantic in six distinct phases [29]: Iberia–Newfoundland, Porcupine–North America, Eurasia–Greenland (conjugate to Rockall), North America–Greenland (Labrador Sea), Eurasia–Greenland (Greenland and Norwegian Sea and Jan Mayen) and North America–Eurasia (Eurasian Basin, Arctic Ocean) (figure 4). The onset of seafloor spreading in the southernmost North Atlantic between Iberia and Newfoundland is heavily debated, with estimates ranging from 149.35 to 112 Ma; older estimates are based on equivocal evidence of magnetic anomaly M21 [119], through to deep sea drilling and seismic refraction studies which suggest dates in the range 128.66–130.2 Ma [120], to stratigraphic studies that suggest dates as young as latest Aptian [121]. Seafloor spreading was certainly initiated by the Mid–Late Albian (110–105 Ma) based on the dating of the sediments overlying tholeiitic basalts at DSDP sites 550 and 551, an Aptian regional unconformity [109], and evidence of magnetic anomaly C34 (83.64 Ma) seaward of this location [122]. The last rifting (Late Cretaceous) phase in the Eurasian basin led to break-up and seafloor spreading; most authors agree that the oldest magnetic anomaly that can be identified is anomaly C25 (approx. 56 Ma), but there are strata landward of these anomalies which suggests that seafloor spreading initiated earlier [29]. True seafloor spreading is believed to have been established by Chron 13 (33 Ma), which coincided with a major reorganization of the Greenland–Eurasian system and cessation of spreading in the Labrador Sea.

As elaborated above, there is clear equivocation over the dating of component phases of the opening of the Atlantic Ocean, but these are perhaps insignificant in comparison with the differences in the timing of separation of regions within the Atlantic. Although continental rifting began in the Triassic and the rudiment of an ocean appears as a consequence of seafloor spreading by the Early Jurassic, there remained links between eastern and western continents until well into the Cenozoic. Thus, the impact of this episode in driving lineage divergence will have spanned at least the interval 247.1–83.6 Ma and potentially longer. For groups with low dispersal ability, like onychophorans and amphibians, the initial phases of continental rifting are likely to have served as great as an agent of lineage divergence as full-scale opening of the ocean on organisms with a greater geographical and ecological range (figure 4). Larger-scale marine barriers would have been required for the fragmentation of flying organisms like insects or birds (although even among those taxa there are notably poorly dispersed lineages), but the aerial distribution of spores and pollen of plants would have mitigated against the impact of the opening Atlantic until the ocean was extensive in both longitude and latitude (figure 4). For instance, in grasses it has been estimated that dispersal events are correlated with ocean width up to a distance of 5000 km, after which they become unpredictable [58], whereas more sessile organisms are likely to show different patterns. Thus, the ecology and dispersal ability of the organisms in question requires different temporal constraints on lineage divergence based on the same tectonic data.

A simplistic view of disjunct distributions being caused by vicariance follows the commonplace assumption that Pangaean organisms exhibited pandemic distributions. While this could be true for some organisms (e.g. the bivalve *Claraia*), it is naive to assume that this will have been generally true for organisms living on a supercontinent that extended from pole to pole with heterogeneous distribution of vegetation, aquatic bodies and mountain ranges forming barriers to dispersal. Rather, it is more likely that different clades were restricted to different regions of Pangaea and, therefore, that divergence often predates continental fragmentation [123,124], direct evidence of which exists for Triassic synaspids and diapsids [125]. Such pseudocongruence between modern and past distributions leads to inaccurate calibrations and, consequently, inaccurate divergence time estimates. In this instance, calibrations based on the opening of the Atlantic will lead to underestimates of the timing of lineage divergence. In other cases, the divergence might considerably postdate the separation of two continents despite a pseudocongruent distribution (e.g. cichlids; [126,127]). Thus, it is imperative that modern distribution patterns are tested for historical veracity by considering the palaeobiogeography of lineages based on the fossil record. For example, it has been argued that placental mammals spread from the southern continents because the earliest branching lineages have an African (Afrotheria) and South American (Xenarthra) modern distribution. However, the earliest records of placentals are from North America and Eurasia, suggesting that they spread into the southern continents only latterly [128–130]. If dispersal ability played a role, it should be anticipated that organisms with low dispersal ability diverge first, while organisms with high dispersal ability diverge later because they are capable of maintaining gene flow over smaller sea barriers for longer time. Dispersal ability might be less important in the case of land bridges that connect once separated, environmentally disparate regions, where environmental changes (e.g. climate: [77]) or biotic interactions (e.g. competition) might be the key factors determining which lineages cross. Dispersal ability might be more important during the break-up of landmasses where regions are initially environmentally similar, and become increasingly dissimilar as they are affected by changes in ocean currents, palaeogeographic position and changed climate, as a consequence of rifting.

## 8. Concluding discussion

When inherent assumptions and attendant errors are considered, calibrations inspired by geological events are no more precise than those based on the fossil record, nor are they any easier to codify. Nevertheless, biogeographic calibrations may be useful since they provide the only effective means of calibrating divergence time analyses that is independent of palaeontological evidence [47,61], a factor that is especially important in groups that lack a coherent fossil record (e.g. soft-bodied parasites [36]) and where secondary calibrations or higher-level dated phylogenies may be the only other alternative for obtaining divergence times [131]. Most importantly, there is no dichotomy in employing fossil and biogeographic calibrations because the age constraints that they impose on divergence time studies are entirely compatible. Indeed, fossil data should be used more widely in informing historical biogeography and refining hypotheses on the impact of geological—and in particular tectonic—events on the geographical range of species, rather than based solely on extant biodiversity [132]. Lastly, geological data are much more readily interpreted for establishing maximum constraints on the age of clades than are palaeontologic data (by using the oldest age of the tectonic event that might have resulted in the current distribution).

Employing biogeographic and fossil calibrations in separate phylogenetic analyses for the same taxon may allow the efficacy and impact of both classes of data in divergence time estimation to be established. Above all, these two approaches provide a means of avoiding circularity, using biogeographic calibrations to assess the efficacy of the fossil record, and fossil calibrations to infer geological history (cf. §6). When all errors are considered, neither approach affords particularly precise time priors. Ultimately, however, it is better to have an accurate timescale of evolutionary history that lacks the precision that we want, than a precise timescale that lacks the accuracy that we need.
